# Methyl Sulfone Blocked Multiple Hypoxia- and Non-Hypoxia-Induced Metastatic Targets in Breast Cancer Cells and Melanoma Cells

**DOI:** 10.1371/journal.pone.0141565

**Published:** 2015-11-04

**Authors:** Joan McIntyre Caron, Jane McIntyre Caron

**Affiliations:** JMCaron, LLC, Thomaston, CT, 06787, United States of America; University of Alabama at Birmingham, UNITED STATES

## Abstract

Metastatic cancer causes 90% of cancer deaths. Unlike many primary tumors, metastatic tumors cannot be cured by surgery alone. Metastatic cancer requires chemotherapy. However, metastatic cells are not easily killed by chemotherapy. These problems with chemotherapy are caused in part by the metastatic cell niche: hypoxia. Here we show that the molecule, methyl sulfone, normalized metastatic metabolism of hypoxic breast cancer and melanoma cells by altering several metabolic functions of the cells. Under hypoxia, methyl sulfone decreased expression of the master regulator of hypoxia, HIF-1α, and reduced levels of the glycolytic enzymes, PKM2, LDHA, GLUT1, the pro-angiogenic protein, VEGF, and the iron-sulfur metabolism molecules, miR-210 and transferrin, all of which promote metastasis. Conversely, methyl sulfone increased levels of ISCU1/2 and ferroportin, proteins associated with iron-sulfur cluster biogenesis and iron homeostasis in normal cells. These data identify methyl sulfone as a multi-targeting molecule that blocks the survival/proliferative effect of hypoxia on metastatic cells and brings normality back to cellular metabolism.

## Introduction

The World Health Organization (WHO) estimates that there were 8.2 million cancer related deaths worldwide in 2012 [1]. To put this number in perspective, the current population of New York City is 8.4 million [2]. In other words, 98% of New York City residents would have died in 2012.

It is well established that 90% of cancer deaths are from metastasis of the primary tumor making the understanding of the metastatic process of utmost concern. In previous work we established that the molecule, methyl sulfone, does not kill cancer cells but instead decreases metastatic phenotypes and increases normal differentiated phenotypes [3].

Methyl sulfone is a small (94 g/mol), water-soluble, non-toxic molecule that was granted GRAS (Generally Recognized As Safe) status by the FDA on July 11, 2007. This molecule is in many of the foods we eat that are considered to be anti-carcinogenic, such as broccoli and cauliflower. Methyl sulfone has been present in our atmosphere, oceanic plankton and vegetation for millions of years.

In the metastatic Cloudman S-91 (M3) melanoma cell line, methyl sulfone induces cell cycle arrest, proper melanocyte structure including melanosome-filled arborization, cellular senescence, and loss of ability to migrate through an extracellular matrix [3]. We demonstrated anticancer activity with methyl sulfone in the metastatic breast cell line, 66cl-4 [4], as well as in cancerous tissue of 17 breast cancer patients [5], again with decreasing metastatic phenotypes and increasing normal phenotypes. Normal breast tissue from the 17 patients retained proper healthy breast structure for at least 90 days in culture in the presence of methyl sulfone. The case has been made that cancerous tumors are wounds that do not heal [6,7]. However, we showed that metastatic and normal breast tissue, 66cl-4 breast cancer cells and M3 melanoma cells carry out proper wound healing in the presence of methyl sulfone, but not in the absence of the molecule.

The issue of mechanism of action of methyl sulfone is a critical question. To begin to understand how methyl sulfone reverses metastatic phenotypes without toxicity we studied the effects of a hypoxic microenvironment on metastatic cells with a focus on cellular metabolism.

Hypoxia plays a significant role in the progression of primary cancer cells to metastasis [8]. Decreased oxygenation of proliferating cancer cells initiates a response for survival. Normal cells, as well as cancer cells, adapt to low oxygen through induction of hypoxia inducible factor (HIF) [9]. HIF-1 is a heterodimer consisting of HIF-1α and HIF-1β subunits. In oxygenated cells, HIF-1α is destined for ubiquitination and degradation. In hypoxia, HIF-1α translocates to the nucleus, dimerizes with HIF-1β and activates gene transcription to support the survival of cancer cells [10]. To date, several genes have been associated with HIF activation. In order to look at a broad range of target genes and the effects of methyl sulfone, we selected targets that have been determined to support metastasis via changes in metabolism. These include HIF-1α [8], angiogenesis, VEGF [11], energy metabolism, PKM2 [12], LDHA [13], GLUT-1 [14], and iron-sulfur metabolism, ISCU1/2 [15], transferrin [16], ferroportin [17], miR-210 [18].

Acute hypoxia is a normal process in cells. It can be caused by extended exercise, inflammation or infection. The hypoxic response reestablishes oxygenation. In cancer cells the hypoxic response continues setting off a cascade of cellular responses through the activation of HIF thereby promoting proliferation and survival of cancer cells. We show here that methyl sulfone significantly reduced HIF-1α expression under hypoxia in metastatic breast and melanoma cells.

As tumors continue to grow, the oxygen partial pressure (pO_2_) drops in tumor cells thereby maintaining a hypoxic phenotype. The cellular response is to initiate angiogenesis. Vascular endothelial growth factor (VEGF) is a direct target of HIF. We show that methyl sulfone down regulated VEGF in the hypoxic environment of metastatic breast cancer cells.

In addition to needing oxygen, cancer cells require an enhanced energy source and a method of obtaining metabolites to synthesize amino acids, nucleic acids, and lipids to support newly accelerated cell growth. The shift in cellular metabolism from oxidative phosphorylation to aerobic glycolysis in the metastatic cell niche [19] is well established and began with the research of Otto Warburg [20]. The Warburg effect is the process in cancer cells whereby cells continue with aerobic glycolysis even when oxygen is present. The current understanding is that aerobic glycolysis allows cancer cells to switch from energy production to the synthesis of metabolites necessary for new cell growth and back again as demands change. We examined three glycolic proteins known to be involved with aerobic glycolysis and metastatic progression.

Pyruvate kinase isoform M1 (PKM1) is the tetrameric isoform that allows glucose in normal cells to enter the mitochondria and begin oxidative phosphorylation. PKM2, the dimeric form, supports the process of aerobic glycolysis. PKM2 is necessary for cancer cells to produce energy and synthesize metabolites for cell proliferation [21]. In contrast, the PKM2 isoform is only present in embryonic cells and cancer cells. In cancer cells, PKM2 levels increase under hypoxia. We show that methyl sulfone significantly decreased the level of PKM2 in two metastatic cell lines under hypoxic conditions.

In aerobic glycolysis the level of lactic acid is high due to diversion of pyruvate away from oxidative phosphorylation and towards production of lactic acid. Lactate dehydrogenase A (LDHA) breaks down lactic acid for energy use in cancerous cells and can be elevated in a hypoxic environment [22]. We show that the level of LDHA was reduced in methyl sulfone-treated breast cancer cells under hypoxia and normoxia.

Because cancer cells have a need for increased glucose for energy, a third glycolic protein comes into play. Glucose transporter 1 (GLUT1) is elevated in cancer cells. The expression of GLUT-1 is controlled by both hypoxia and diminished oxidative phosphorylation [14]. Over expression of GLUT-1 is associated with a greater likelihood of metastasis in lung cancer [23], cervical cancer [24] and colorectal cancer [25]. GLUT-1 is expressed at a reduced level in normal cells compared to cancer cells. We show that methyl sulfone deceased the level of GLUT-1 in metastatic, hypoxic cancer cells.

The next metabolic group we studied to gain insight into the mechanism of action of methyl sulfone is iron-sulfur metabolism. It has been well established that HIF directly targets micro-RNA-210 (miR-210) [26]. The expression of miR-210 has a cascading effect on the metabolism of iron and sulfur, two essential elements for viability. With the expression of miR-210, iron-sulfur cluster scaffold proteins (ISCU1/2) are repressed [27]. This repression causes a disruption of iron-sulfur cluster (Fe-S cluster) biogenesis in the mitochondria [28]. The failure of Fe-S cluster biogenesis causes an increase in transferrin [29]. Cancer cells crave iron; the increase in transferrin and decrease in ferroportin [30,31] leads to an increase in the labile iron pool, providing additional iron to support cancer cell proliferation.

Fe-S cluster biogenesis begins in the mitochondria and once formed, clusters exit to the cytosol. Mitochondrial iron-overload and genomic instability result from the failure of Fe-S cluster biogenesis [32]. Ferroportin is the only know iron efflux pump protein and is necessary as part of tightly controlled iron homeostasis. Studies have demonstrated a correlation between decreased cellular ferroportin and metastatic progression [33]. We show that methyl sulfone decreased miR-210 and transferrin, and increased ISCU1/2 and ferroportin.

Our results identify methyl sulfone as a multi-targeting small molecule that blocks the survival/proliferative effect of hypoxic and non-hypoxic promoters of metastatic phenotypes and brings normality back to cellular metabolism.

## Materials and Methods

### Materials

Methyl sulfone was purchased from Fluka/Sigma Co., St. Louis, MI.

### Cell culture

The murine 66cl-4-cell line is a subclone of the metastatic breast cancer cell line 4T1 [34–36]. The 66cl-4 cells were grown in Dulbecco’s modified Eagle’s medium (DMEM) supplemented with 7% fetal bovine serum (Invitrogen, Inc., Eugene, OR). M3 melanoma cells, a melanin-producing cell line, are a subclone of murine Cloudman S-91 melanoma cells. Using the method of Slominski et al. [37] we showed that control and methyl sulfone-treated M3 melanoma cells produced equal amounts of melanin. M3 melanoma cells were grown in RPMI supplemented with 7% fetal bovine serum. Human CCRF-CEM T-cell acute lymphocytic leukemia cells were isolated from the peripheral blood of a 4-year old girl. CEM leukemic lymphocytes were grown in modified Eagle’s medium (MEM) with Earle’s salts, L -glutamate and 7.5% fetal bovine serum. All cell lines were passaged twice per week. Control cells (no methyl sulfone) and 200 mM methyl sulfone-treated cells were cultured under normoxia (21% O_2_, 5% CO_2_) or hypoxia (0.1% O_2_, 5% CO_2_) at 37°C.

### Immunoblot analysis

For immunoblot analysis, antibodies to ferroportin-1, GLUT1, HIF-1α, ISCU1/2 and PKM2 were purchased from Santa Cruz Biotechnology, Santa Cruz, CA. HIF-1α antibody was purchased from Thermo Fisher Scientific, Pittsburgh, PA. Antibody to LDHA and YAP was purchased from Cell Signaling Technology, Danvers, MA. Antibody to tubulin was purchased from Sigma-Aldrich. Secondary HRP-antibodies were purchased from GE/Amersham, Pittsburgh, PA, and Santa Cruz Biotechnology.

Because control cells grow faster than methyl sulfone-treated cells, control cells were plated at a density of 5X10^5^ cells/ml and cells to be treated with methyl sulfone were plated at a density of 1X10^6^ cell/ml in 100 mm culture dishes. After 24 hours in culture, methyl sulfone (200 mM) was added to the higher density cells. Media was changed every 48 hours and cells were examined daily by phase contrast microscopy. After 3–7 days, when methyl sulfone-treated cells became contact inhibited, half of the control cells and half of the methyl sulfone-treated cells were incubated under hypoxic conditions using a Billups-Rothenberg modular incubation chamber, Del Mar, CA. Remaining cells continued to be incubated under normoxia. After six hours, cells were placed on ice and immediately lysed [38] in the presence of protease inhibitors (SigmaFast protease inhibitor cocktail, Sigma-Aldrich, Inc.). After 10 min on ice, samples were boiled for 5 min, centrifuged (14,000Xg, 4°C) for 10 min, and the supernatants were transferred to fresh tubes followed by a BioRad DC protein assay (BioRad, Inc., Richmond, CA). Lysates were stored at -80°C.

Four protein samples were analyzed: Control-Normal (CN), Control-Hypoxia (CH), Methyl sulfone-Normoxia (MN) and Methyl sulfone-Hypoxia (MH). Equal amounts of protein (15μg) were run on 4–15% or 8–16% BioRad mini protein gels. Antibodies to YAP and tubulin protein were used as loading controls. In addition, duplicate gels were stained with Coomassie Blue followed by ImageJ analysis to confirm equal protein load between samples. For protein sizing and gel orientation, three lanes containing one of two unique cocktails of colored molecular weight markers (Sigma Color-burst markers and BioRad kaleidoscope markers) framed sample lanes. Proteins were transferred to 0.2μm nitrocellulose and blots were incubated with primary and secondary antibodies according to the manufacturers. Bands were detected using the Amersham ECL Prime detection reagent and the Syngene G:Box imaging system, Frederick, MD. Bands were scanned using ImageJ. Immunoblots were performed at least in duplicate. Lysates from at least three experiments were isolated from each cell line. Immunoblots shown here were from one experiment of each cell line.

### miR-210 analysis

Cells were grown in 100 mm plates, treated with and without 200 mM methyl sulfone under normoxic and hypoxic conditions as described for Immunoblot Analysis. Plates were placed on ice, cells were lysed with TRIzol (Life Technologies, Eugene, OR) and RNA was isolated as described by the manufacturer. RNA samples were sent to LC Sciences, Houston, TX, on dry ice. LC Sciences performed RNA quality tests and then analyzed RNA samples with real time QPCR for relative levels of miR-210-3p and miR-210-5p using their MicroRNA Service. Four RNA samples were analyzed: Control-Normoxia (CN), Control-Hypoxia (CH), Methyl sulfone-Normoxia (MN) and Methyl sulfone-Hypoxia (MH). Each of the four samples was tested for miR-210-3p and miR-210-5p in triplicate. Data were presented as six comparisons of the four samples: CN–CH, MN–MH, CN–MN, CH–MH, CN–MH and CH–MN.

### Immunofluorescence of tubulin

Adherent cells (66cl-4 breast cancer cells and M3 melanoma cells) were grown on glass coverslips and non-adherent cells (CEM leukemic lymphocytes) were grown in petri dishes. The latter cells were centrifuged briefly, and the supernatant discarded. All cell samples were fixed in a microtubule-stabilizing buffer [39]. Tubulin/microtubules were visualized with a mouse anti-α-tubulin antibody (Sigma-Aldrich, Inc.) and a Rhodamine-labeled secondary antibody (Molecular Probes, Eugene, OR) using an Axioplan CCD microscope equipped with a 63X~1.4 NA Plan Apo oil immersion objective via Metamorph image acquisition and analysis software (Universal Imaging Corp., Downington, PA).

### Palmitoylation of tubulin

Non-radioactive analysis of palmitoylation (Biotin Switch Assay) was performed using the methods of [40] and [41]. Briefly, M3 melanoma cells were plated at 1X10^5^ cells/ml in 100 mm dishes. After 24 hours, 200 mM methyl sulfone was added to half of the plates. After 10 min, plates were placed on ice, washed twice with ice-cold phosphate-buffered saline (PBS) and cells were scraped into ice-cold 10 mM sodium phosphate, pH 7.4, 2 mM EGTA, 0.32 M sucrose, 50 mM N-ethylmaleimide (NEM, Sigma-Aldrich), and protease inhibitors. Cells were passed through 25 gauge needles to break cells, and then incubated on ice for six hours to bind NEM to free sulfhydryl groups. Sodium dodecyl sulfate (SDS) was added to a final concentration of 1%. Lysates were sonicated on ice for 30 seconds and centrifuged at 4°C for 15 min. Supernatants were transferred to fresh tubes and Triton X-100 was added to a final concentration of 1%.

Protein G Dynabeads (Life Technologies, Inc.) were prepared by incubation with anti-α-tubulin antibody (DMA1, Abcam, Inc., Cambridge, MA) according to the procedure described by Life Technologies, Inc. Dynabeads/α-tubulin antibody mixture was incubated with lysates overnight at 4°C on a rotator. Dynabead-protein complexes were then incubated with palmitate hydrolysis buffer for two hours at room temperature to remove palmitate from proteins with hydroxylamine (Sigma-Aldrich, Inc.), and to substitute EZ-Link (R) BMCC-Biotin (Thermo Fisher Scientific Co.) for the removed palmitate. Palmitate hydrolysis buffer was 1 M hydroxylamine, 80 μM EZ-Link (R) BMCC-Biotin, 10 mM sodium phosphate, pH 7.4, 2 mM EGTA and protease inhibitors. In half the samples, 1M TRIS, pH 7.4 was substituted for hydroxylamine as a negative control.

Dynabead-protein complexes were washed, re-suspended in Laemmli sample buffer, boiled for 5 min, and run on duplicate BioRad 8–16% mini protein gels. Immunoblots were processed as described above using anti-biotin antibody (Abcam, Inc.) as a measure of palmitoylated α-tubulin levels, and anti-α-tubulin antibody (Abcam, Inc.) as a measure of total α-tubulin levels. Bands were scanned using ImageJ. To calculate relative levels of palmitoylated α-tubulin in control and methyl sulfone-treated melanoma cells, band intensities in TRIS samples were subtracted from band intensities in hydroxylamine samples.

### In vitro microtubule assembly

In vitro microtubule assembly was measured using an HTS-tubulin polymerization assay kit (Cytoskeleton, Inc., Denver, CO) with 96 half well plates. Tubulin was used at 40μM. Methyl sulfone was used at 0 (control), 0.8μM, 4μM, 40μM (equi-molar to tubulin), 400μM and 2mM. All samples were run in duplicate. Taxol (4μM) alone was also used as a control, and compared to Taxol (4μM) in the presence of 4μM methyl sulfone. Assembly buffer contained 1mM GTP, but no glycerol. Microtubule assembly was measured at BioTek, Inc., Winooski, VT, using a BioTek Synergy H4 reader with Gen5 data analysis software. The reader was set at 37°C, 340nm and a kinetic loop to read samples at one min intervals for 90 min. Initial lag time, Vmax, area under curve (AUC) and change in OD_340_ over 90 min were determined.

## Results

### Methyl sulfone reduced levels of HIF-1α in metastatic breast cancer and melanoma cells under hypoxia

**Fig 1:**
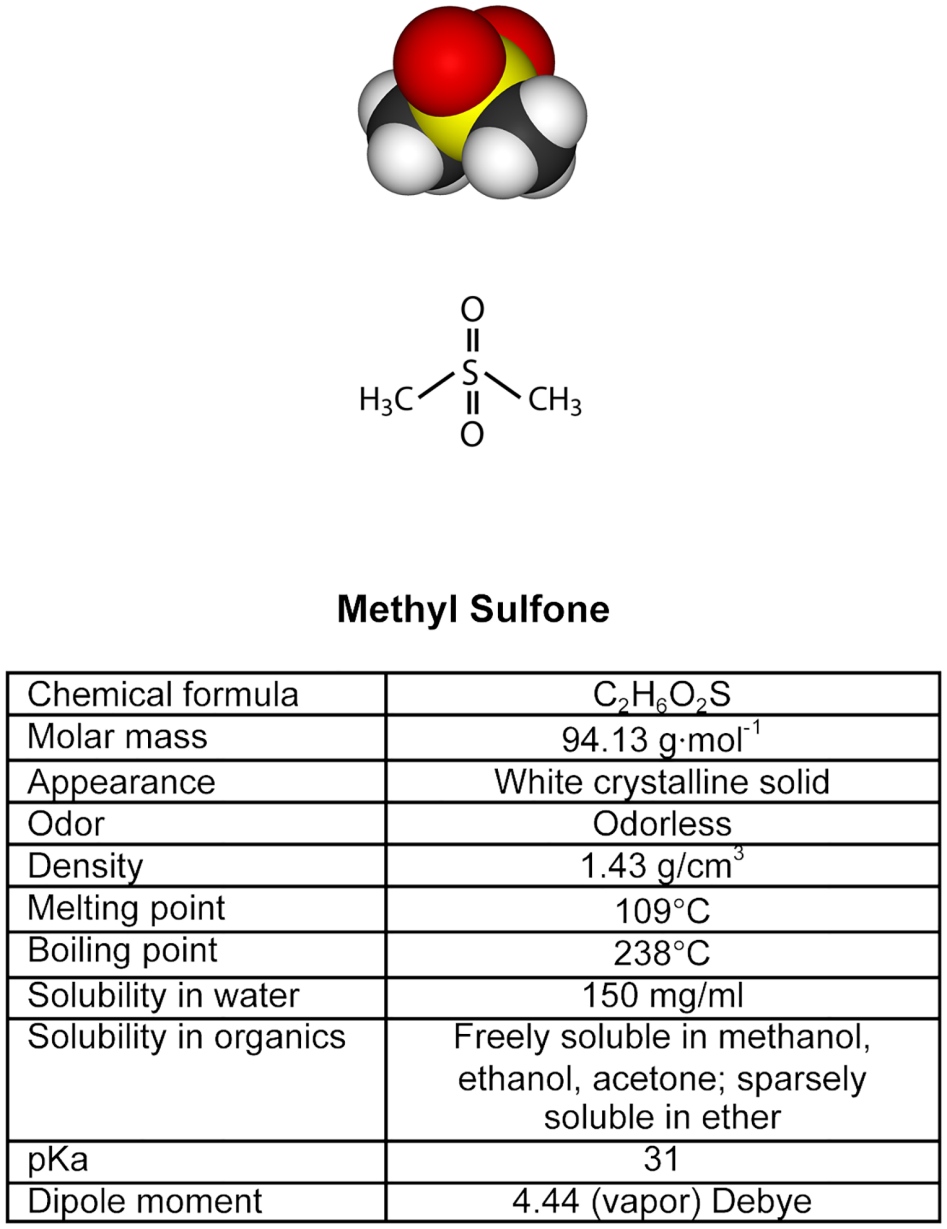
The chemical structure and properties of methyl sulfone.

**Fig 2:**
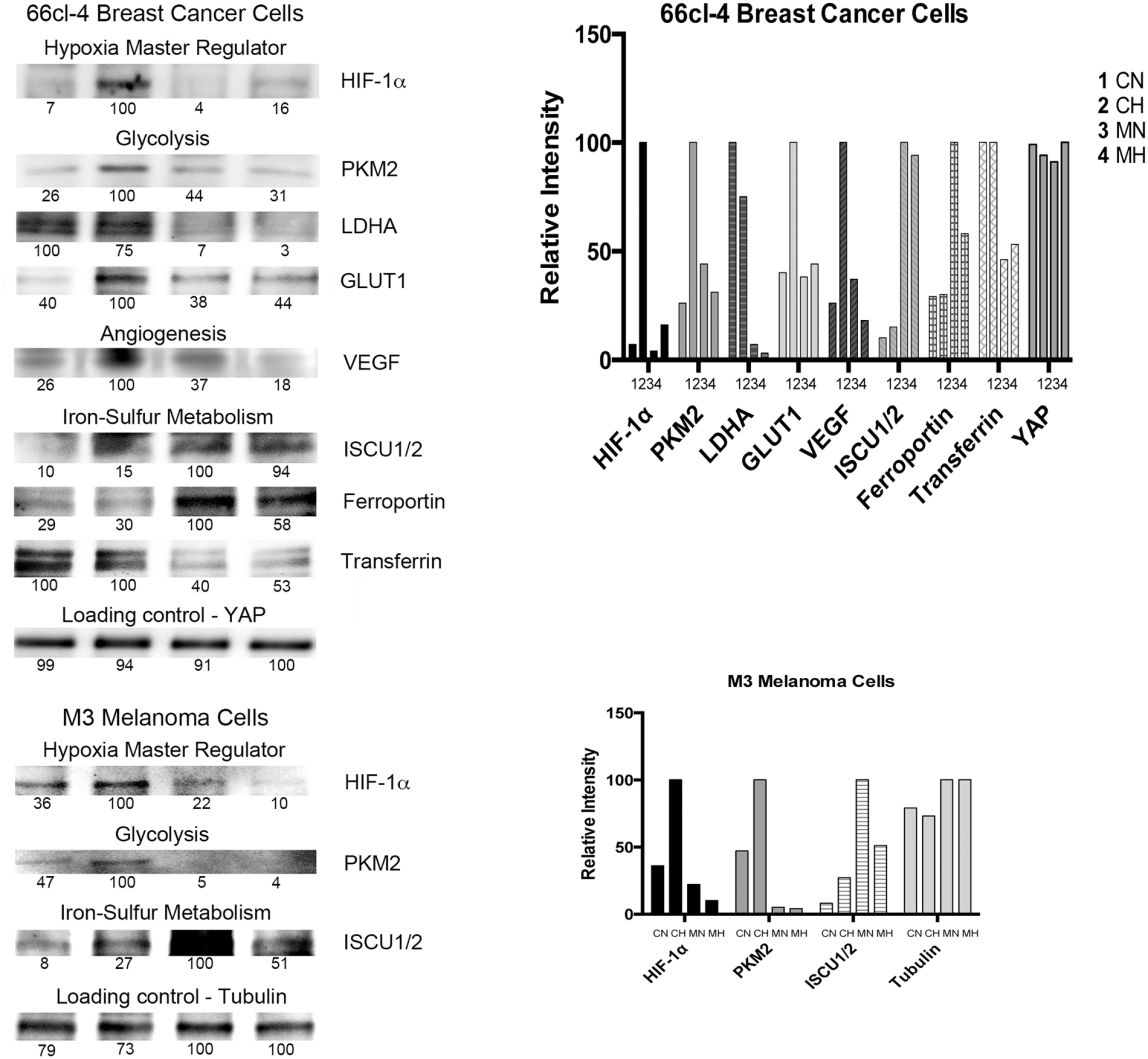
Effect of methyl sulfone on hypoxia-dependent and -independent expression of proteins in metastatic cells. 66cl-4 breast cancer cells and M3 melanoma cells were grown under normoxic conditions in the presence and absence of methyl sulfone. When cells became confluent, half of the control plates and methyl sulfone-treated plates were placed under hypoxia. The remaining plates continued incubation under normoxic conditions. This led to four samples: control-normoxia (CN), control-hypoxia (CH), methyl sulfone-normoxia (MN), and methyl sulfone-hypoxia (MH). After six hours, cells were immediately put on ice, lysed, and prepared for immunoblot analysis. ImageJ determined relative levels within each set of the four samples. The most intense band was set at 100. Equal loading of protein was assessed by immunoblot analysis of YAP protein in 66cl-4 lysates and tubulin protein in M3 lysates. The immunoblots shown in Fig 2 were obtained from one lysate experiment for each cell line.

Metastatic breast cancer cells, 66cl-4 mouse breast cancer cell line [4] were cultured for 24 hours. Methyl sulfone (Fig 1) was added to half the plates to a final concentration of 200 mM, and cells were cultured for an additional 7 days during which contact inhibition was established in both cell types. Half of the control cells (no methyl sulfone) and half of the methyl sulfone-treated cells were then incubated under hypoxic conditions (0.1% O_2_). The remaining cells were cultured under normoxic conditions (21% O_2_). After six hours cells were lysed. Relative HIF-1α levels in cell extracts were determined by immunoblot analysis. In the metastatic breast cancer cells, HIF-1α levels increased from 7% (CN) to 100% (CH) (a 14-fold increase) when control-normoxia (CN) cells were compared to control-hypoxia (CH) cells (Fig 2). Conversely, HIF-1α levels only increased in methyl sulfone-treated-normoxia (MN) cells in response to hypoxia (MH) from 4% (MN) to 16% (MH) (a 4-fold increase). Comparison of HIF-1α levels in CH cells with MH cells demonstrated a 6-fold higher increase in CH, from 16% (MH) to 100% (CH).

In metastatic M3 melanoma cells, hypoxia induced a 3-fold increase in control cells (36% to 100%; CN to CH), but no increase in methyl sulfone-treated cells. In fact, in methyl sulfone-treated cells, HIF-1α levels were lower under hypoxia when compared to normoxia (22% to 10%; MN to MH) (Fig 2). Comparison of HIF-1α levels in CN cells versus MH cells demonstrates a 10-fold higher increase in CN cells (10% to 100%; MH to CH). The presence of melanin can alter metabolism [37,42] and up-regulate HIF-1α levels [37]. However, M3 melanoma cells are a melanin-producing cell line, and both control cells and methyl sulfone-treated cells produce equal amounts of melanin, negating potential differentiating effects of melanin on HIF-1α levels.

### Under hypoxia methyl sulfone reduced levels of glycolytic enzymes associated with metastatic progression

The levels of glycolytic enzymes, PKM2, LDHA and GLUT1, increase in response to increased HIF-1α [10]. We therefore tested levels of these enzymes in control and methyl sulfone-treated 66cl-4 metastatic breast cancer cells under normoxic and hypoxic conditions (Fig 2). PKM2 increased in CH cells (to 100%), but levels were relatively low in CN (26%), MN (44%) and MH (31%) cells. Comparison PKM2 levels of CH versus MH cells under hypoxia demonstrated a greater than 3-fold increase in CH cells (31% to 100%; MH to CH).

In control metastatic M3 melanoma cells PKM2 levels increased under hypoxia (47% to 100%; CN to CH) (Fig 2). However, in methyl sulfone-treated cells PKM2 levels were low under normoxia (5%), and did not increase under hypoxia (4%). As with HIF-1α, methyl sulfone-treated cells under normoxia had higher levels of PKM2 than methyl-sulfone-treated cells under hypoxia.

In metastatic 66cl-4 breast cancer cells, LDHA levels were highest in CN (100%) and CH (75%) cells, but expression of LDHA in control cells was not hypoxia-dependent (Fig 2). Levels of LDHA were lowest in MN (7%) and MH cells (3%); these levels were also not hypoxia-dependent. Comparison of CH cells and MH cells under hypoxia demonstrated a 25-fold increase of LDHA levels in CH cells (3% to 100%; MH to CH).

In metastatic breast cancer cells, GLUT1 levels were highest in CH cells (100%), and low in CN (40%), MN (38%) and MH (44%) cells (Fig 2). These data demonstrate that expression of GLUT1 in control cells was hypoxia-dependent, but expression of GLUT1 in methyl sulfone-treated cells was hypoxia-independent. These data also demonstrate that under normoxia cells treated with methyl sulfone maintained similar GLUT1 levels compared to control cells. Comparison of GLUT1 levels in CH cells versus MH cells demonstrated a greater than 2-fold increase in CH cells (44% t0 100%; MH to CH).

### Under hypoxia methyl sulfone reduced levels of the angiogenic protein, VEGF, associated with metastatic progression

The angiogenic protein, VEGF, increases significantly in many types of tumors that are hypoxic and progressing towards metastasis [43]. In metastatic 66cl-4 breast cancer cells we found that VEGF levels increased in CH cells (100%) compared to CN cells (26%) as expected, but VEGF levels remained low in MN (37%) and MH (18%) cells (Fig 2). Hypoxia increased VEFG levels in control by 4-fold, but reduced VEGF levels in methyl sulfone-treated cells 2-fold. Comparison of VEGF levels in CH cells versus MH cells demonstrated a greater than 5-fold increase in CH cells (18% to 100%; MH to CH).

### Under hypoxia methyl sulfone reduced iron transport protein transferrin associated with metastatic progression

Metastatic cancer cells often contain increased levels of proteins that effect iron homeostasis and support proliferation. One of these proteins is transferrin [44], which supports iron transport and is up regulated in cancer cells to meet increased iron demands. We examined levels of transferrin in metastatic breast cancer CN, CH, MN and MH cells. Transferrin levels were highest in CN (100%) and CH cells (100%), and lowest in MN (46%) and MH (53%) cells (Fig 2). Expression of transferrin in control and methyl sulfone-treated cells was not hypoxia-dependent. Comparison of transferrin levels in CH cells versus MH cells demonstrated a greater than 2-fold increase in CH cells (53%% to 100%; MH to CH).

### Under hypoxia methyl sulfone increased proteins involved in Fe-S cluster biogenesis and iron homeostasis found in normal cells

Metastatic cells often lose their ability to make Fe-S clusters, structures that are required for normal cell processes, including iron homeostasis. This loss of ability is due to decreased levels of ISCU1/2, a direct target of miR-210. The down-regulation of ISCU proteins results in the up-regulation of transferrin receptor 1. Ferroportin is an iron efflux protein whose down regulation has been associated with poor prognosis [17].

Examination of ISCU1/2 in metastatic breast cancer cells revealed the highest levels in MN (100%) and MH (94%) cells, and the lowest levels in CN (10%) and CH (15%) cells (Fig 2). Expression of ISCU1/2 was not hypoxia-dependent. Comparison of ISCU1/2 in control cells and methyl sulfone-treated cell revealed an approximately 8-fold higher level of expression in methyl sulfone-treated cells.

Similarly, in metastatic melanoma cells the highest levels of expression of ISCU1/2 were found in MN (100%) and MH (51%) cells, and lowest levels in CN (8%) and CH cells (27%) (Fig 2). As with breast cancer cells, expression of ISCU1/2 was not hypoxia-dependent. Comparison of ISCU1/2 levels in control cells and methyl sulfone-treated cells revealed an approximately 2-fold increase in methyl sulfone-treated cells.

In metastatic 66cl-4 breast cancer cells, the highest levels of expression of ferroportin were found in MN (100%) and MH (58%) cells compared to CN (29%) and CH (30%) cells. Expression of ferroportin in methyl sulfone-treated metastatic breast cancer cells decreased 1.7-fold under hypoxia, but expression of ferroportin in control cells increased under hypoxia by 9-fold. Importantly, comparison of ferroportin levels in control cells and methyl sulfone-treated cells revealed an approximately 2-fold increase in methyl sulfone-treated cells (30% to 58%; CH to MH).

### Under hypoxia methyl sulfone decreased levels of miR-210-3p and miR-210-5p, two miRNAs associated with metastatic progression

**Fig 3:**
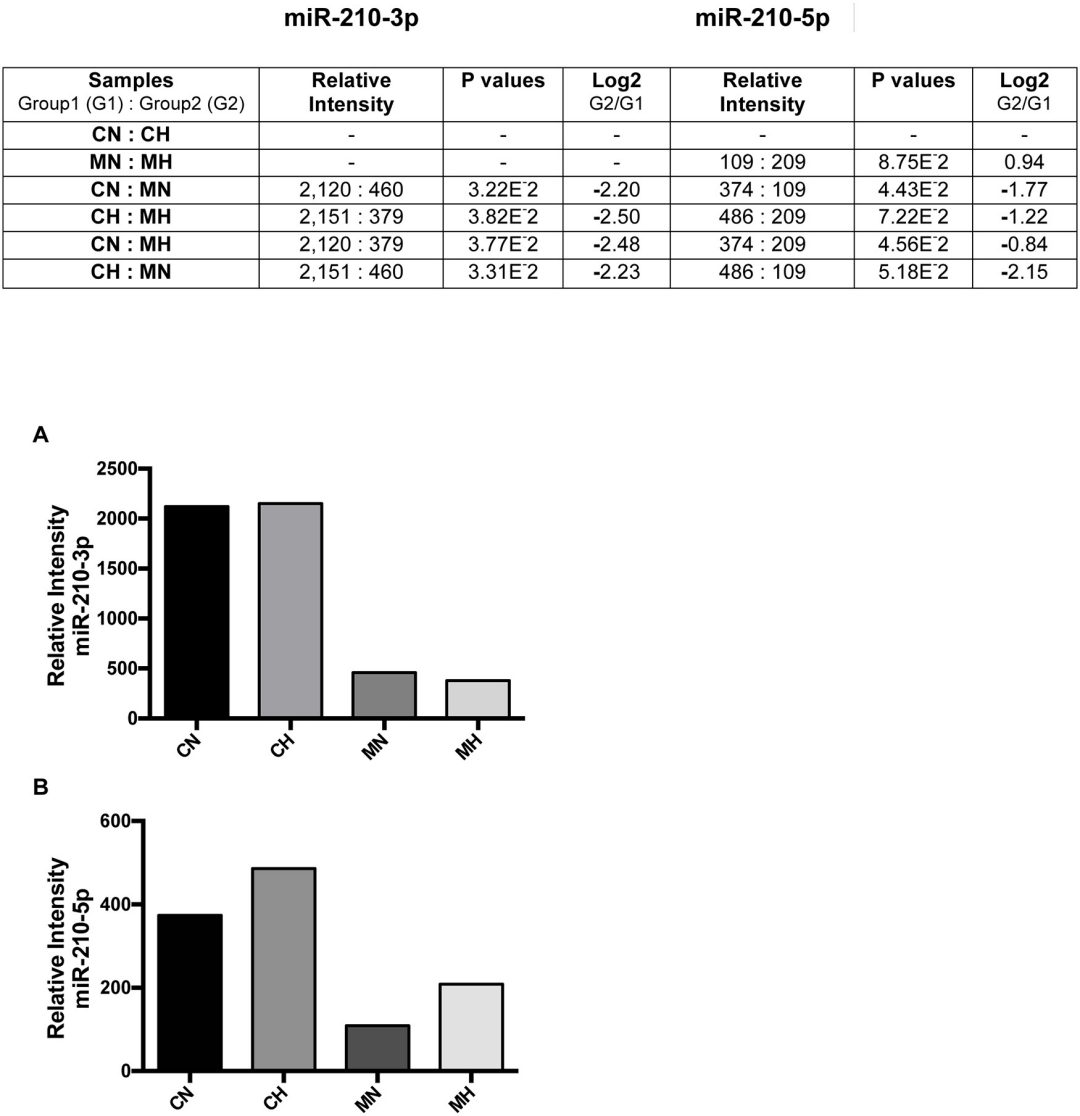
Effect of methyl sulfone on metastatic induction of miR-210-3p and miR-210-5p. (A) 66cl-4 metastatic breast cancer cells were grown to confluence in the presence and absence of methyl sulfone. At confluence, half of the control plates (no methyl sulfone) and half of the methyl sulfone-treated plates were placed under hypoxia at 37°C. The remaining plates continued incubation under normoxic conditions. After six hours cells were solubilized in TRIzol to isolate RNA. Relative levels of miRNA-210-3p and miR-210-5p were determined by microarray analysis and real time QPCR, in triplicate. Six pairwise comparisons of the four samples for each miR are shown (A) along with Log2 differences between pairs. (B) Relative levels of individual miR-210 were determined.

Increased levels of the microRNAs, miR-210-3p and miR-210-5p, are associated with metastatic progression and poor survival [45]. These miRNAs block the production of Fe-S clusters required for normal cell physiology. Therefore, we examined the effect of methyl sulfone in the presence and absence of hypoxia on levels of miR-210-3p and miR-210-5p (Fig 3). In metastatic 66cl-4 breast cancer cells we found that the level of miR-210-3p was highest in CN (2,120; see Fig 3) and CH (2,151) cells, and lowest in MN (460) and MH (379) cells. We found no significant differences in the level of miR-210-3p between CN and CH cells, or between MN and MH cells suggesting that expression of miR210-3p in this cell line was not hypoxia-dependent. Similar results were found with miR-210-5p, although signal intensities were generally lower than in miR-210-3p. In addition, expression of miR-210 5p showed some hypoxia-dependence.

### Methyl sulfone caused rapid depolymerization of microtubules, a concomitant increase in palmitoylated tubulin levels, followed by re-assembly of tubulin in microtubules

**Fig 4:**
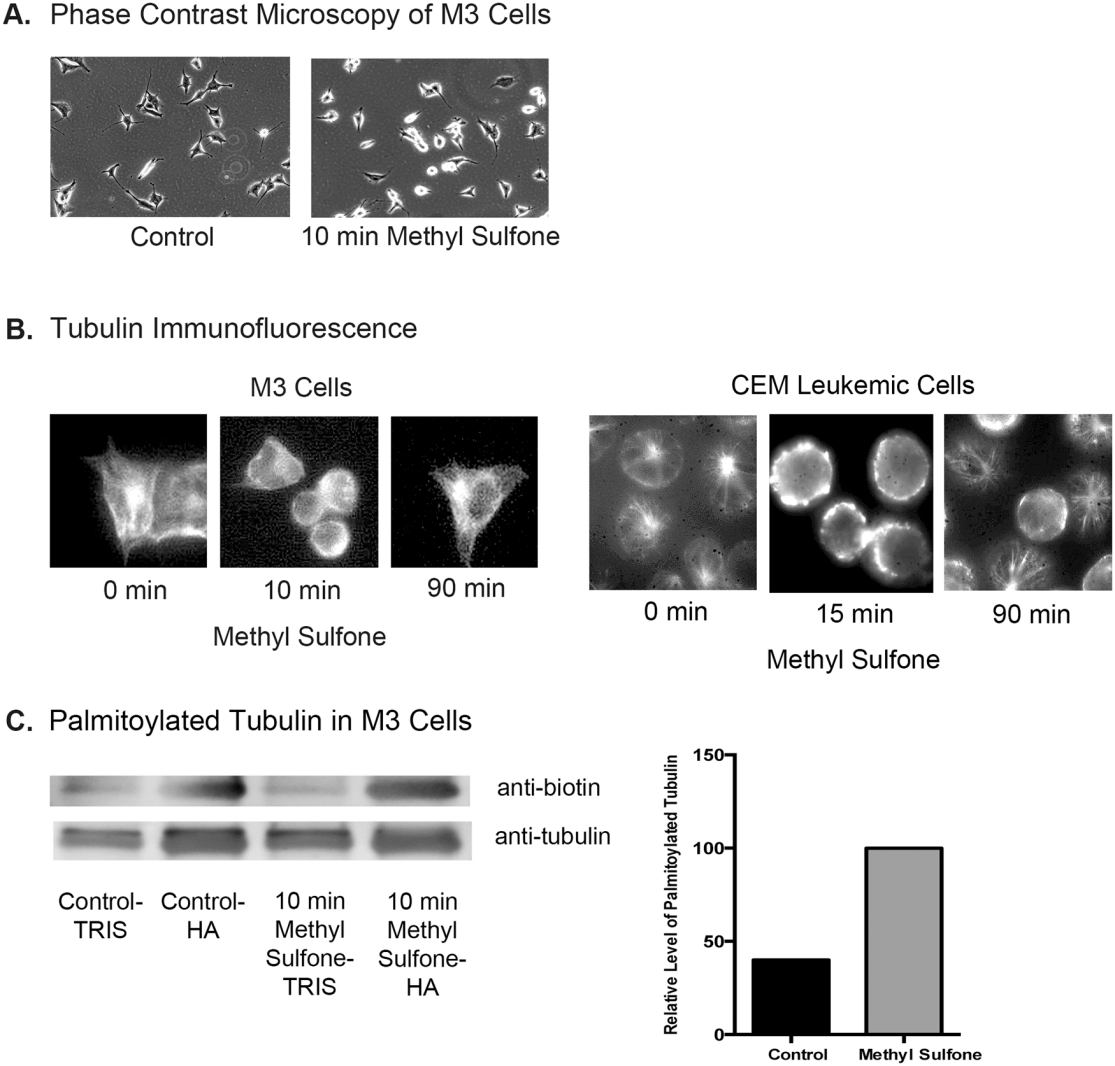
Effect of methyl sulfone on microtubules and tubulin. (A) M3 melanoma cells were incubated in the presence and absence of methyl sulfone. After ten minutes, cells were photographed by phase contrast microscopy using a 10X objective. (B) M3 melanoma cells and CEM leukemic lymphocytes were plated. After 24 hours 200 mM methyl sulfone was added to the cells. At 0 time (immediately before adding methyl sulfone), 10 minutes (M3 cells), 15 minutes (CEM cells) and 90 minutes, cells were processed for immunofluorescence microscopy of microtubules and tubulin. Scale bars are 20 μm. (C) M3 cells were plated. After 24 hours 200 mM methyl sulfone was added. After 10 minutes, cells were lysed and processed for detection of palmitoylated tubulin as described in Methods. Immunoprecipitated tubulin (+/-) biotin was subjected to gel electrophoresis and immunoblot analysis using anti-biotin and anti-tubulin antibody. Relative levels of palmitoylated tubulin were determined with ImageJ.

Microtubules are required to transport the master transcriptional activator, HIF-1α, from the cytosol into the nucleus [46]. Since methyl sulfone blocked HIF-1α accumulation, we examined the effect of methyl sulfone on microtubules. Within 10 minutes of adding methyl sulfone, M3 melanoma cells rounded up (Fig 4). This result was also seen with the 66cl-4 breast cancer cells, suggesting that methyl sulfone may have a general effect on microtubules. Microtubules were visualized by immunofluorescence in M3 melanoma cells and another cell line, CCRF-CEM, a human T-cell leukemic cell line [47]. Time points were immediately before treatment of cells with methyl sulfone, 10–15 minutes after adding methyl sulfone, and 90 minutes later. In both the M3 melanoma cells and CEM leukemic lymphocytes, methyl sulfone induced massive microtubule disassembly within 10–15 minutes in virtually all cells. Either depolymerized tubulin or extremely short microtubules below the resolution of the microscope were visualized along the inside of the plasma membrane. However, after 90 minutes tubulin was no longer on the plasma membrane, but instead had re-assembled into microtubules, demonstrating that cells were not killed by the massive methyl sulfone-induced microtubule depolymerization.

The presence of tubulin on the plasma membrane suggests that tubulin may have been post-translationally modified by palmitoylation [47–50], a reversible modification that drives proteins to membranes. Therefore, we examined whether methyl sulfone induced palmitoylation of tubulin. M3 melanoma cells were incubated with and without methyl sulfone for 10 minutes. Cells were lysed and levels of palmitoylated tubulin were determined as described in Methods. As shown in Fig 4, treatment of M3 cells with methyl sulfone for 10 minutes resulted in a greater than 2-fold increase in the level of palmitoylated tubulin.

The rapid depolymerization of microtubules in cells treated with methyl sulfone followed by re-assembly of microtubules suggests that methyl sulfone has a direct and/or indirect effect on microtubule assembly. To determine whether methyl sulfone has a direct effect on microtubule assembly, we examine microtubule assembly in vitro. Methyl sulfone (0 (control), 0.8μM, 4μM, 40μM, 400μM, 2mM) was added to microtubule protein (40μM) under assembly conditions (37°C, 1mM GTP). Taxol (4μM) was used as a second control. We found no evidence of a direct effect of methyl sulfone on microtubule assembly. There were no differences in initial lag time, Vmax, AUC, or change in OD_340_ over 90 min. In addition, methyl sulfone had no effect on Taxol-induced microtubule assembly. See S1 Table: In vitro microtubule assembly in the presence and absence of methyl sulfone. In S1 Table are data for parameters of in vitro microtubule assembly in the presence and absence of methyl sulfone and Taxol including lag time, Vmax, AUC and change in OD_340_ over 90 min.

### Metastatic cells maintained and formed methyl sulfone-induced contact inhibition under hypoxia

One of the effects of methyl sulfone on metastatic cells is induction of contact inhibition, a phenotype associated with normal cells [3]. We next examined whether metastatic cells maintained methyl sulfone-induced contact inhibition under hypoxia. Metastatic 66cl-4 breast cancer cells and metastatic M3 melanoma cells were cultured for 24 hours. Methyl sulfone (200 mM) was added to half the plates, and cells were cultured for an additional 7 days during which contact inhibition was established in both cell types. Half of the control cells (no methyl sulfone) and half of the methyl sulfone-treated cells were then incubated under hypoxic conditions for 6, 24 or 48 hours. The remaining cells were cultured under normoxic conditions. At several time points cells were examined by phase contrast microscopy to assess the presence or absence of contact inhibition.

**Fig 5:**
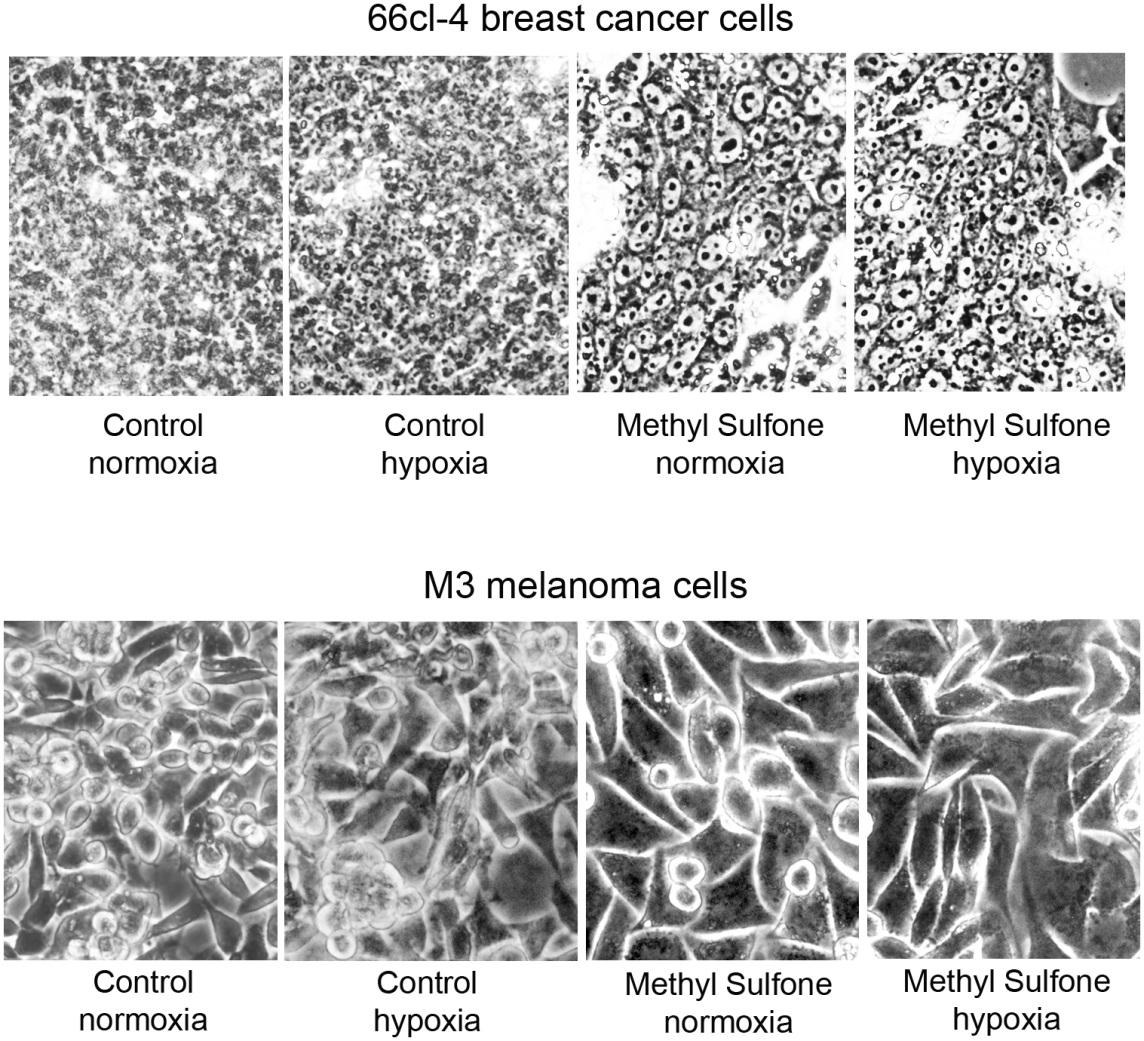
Effect of hypoxia on methyl sulfone-induced contact inhibition in metastatic cells. 66cl-4 breast cancer cells and M3 melanoma cells were grown in the presence and absence of methyl sulfone. At confluence, half of the control plates and half of the methyl sulfone-treated plates were placed under hypoxia. Remaining plates continued incubation under normoxic conditions. After six hours cells were photographed using phase contrast microscopy with a 10X objective.

Shown in Fig 5 are control and methyl sulfone-treated cells in hypoxia for 6 hours. In both breast cancer and melanoma cell types, contact inhibition was maintained in hypoxic cells treated with methyl sulfone. In control cells of both cell lines, no contact inhibition was apparent. Instead, cancer cells grew into a thick mat of overlapping cells under normoxia and hypoxia. Similar results were found when cells were incubated under hypoxia for 24 and 48 hours. These data suggest that under hypoxia methyl sulfone-treated cells were able to resist reversion from contact inhibition found in normal cells to uncontrolled proliferation found in metastatic cells.

Finally, we wanted to address two questions. First, would metastatic cells form contacted-inhibited monolayers while under hypoxia? Second, would metastatic cells become resistant to methyl sulfone after two rounds of hypoxia treatment? We knew from our previous studies [3–5] that the effect of methyl sulfone on contact inhibition was reversible during the first 2–3 weeks in methyl sulfone. After 2–3 weeks in the presence of methyl sulfone, cells became irreversibly contact-inhibited even if methyl sulfone is removed from the media. Therefore, we designed these experiment to take place during the first 2–3 weeks in methyl sulfone when contact inhibition was still reversible.

Metastatic 66cl-4 breast cancer cells were plated and cultured for 24 hours. Methyl sulfone was added to half of the plates to induce contact inhibition. Once contact inhibition had formed, half of the methyl sulfone plates and half of the control plates were placed under hypoxia for 24 hours. Remaining plates were left under normoxia. After 24 hours, methyl sulfone plates under hypoxia and normoxia were switched to control media to cause reversal of contact inhibition, and all plates were incubated under normoxia. After 24 hours, phase contrast microscopy confirmed that MN and MH were no longer contact inhibited. MN and MH cells were placed back into methyl sulfone media and all four samples (CN, CH, MN and MH) were placed back under hypoxia. This was the first round of hypoxia for CN and MN cells, and the second round of hypoxia for CH and MH cells. After 24 hours, phase contrast microscopy was used to assess the cells. CN cells were small, crowded plies of cells. CH cells were small and formed crowded piles of cells. Both MN and MH cells formed a monolayer of contact-inhibited cells. These data demonstrate that cells became contact-inhibited while under a hypoxic environment and that cells do not become resistant to methyl sulfone after two rounds of hypoxia.

## Discussion

### Is hypoxia relevant to melanoma tumors of the skin or to melanoma and breast cancer metastases to the lung, one of the most common sites of metastasis for these types of tumors?

The oxygen levels in blood cells and tissues vary, but pO_2_ is generally 38 mmHg or greater [51]. In the epidermis of the skin, where melanocytes reside, pO_2_ is approximately 35 mmHg. If a melanoma tumor in the skin grows greater than 1 mm in diameter, pO_2_ in the center of the tumor will decease to approximately 7.6 mmHg. This melanoma tumor is now hypoxic. Similarly, if the melanoma metastasizes to the lung, pO_2_ in the lung tumor will decrease to approximately 7.6 mmHg as soon as the tumor grows greater than 1 mm in diameter [52]. The metastatic tumor of the lung then becomes hypoxic. The same chain of events is true for metastatic breast cancer in the lung [53]. Finally, a primary lung tumor will also become hypoxic if the tumor grows to greater than 1 mm in diameter. In support of these conclusions, the master transcriptional regulator of hypoxia, HIF, is localized to the center of tumors in primary melanoma [52] and metastasized breast cancer to the lung [53]. Thus, the effects of hypoxia on metastatic breast cancer and metastatic melanoma remain a major concern.

The presence of hypoxic tumor tissue in patients is associated with poor outcomes [54, 55, 56]. However, we show here that in the presence of hypoxia methyl sulfone reversed and maintained normal phenotypes in metastatic cells. For example, we showed previously and here that methyl sulfone induced contact inhibition, a classic hallmark of normal cells, under normoxia and hypoxia. By deduction, the establishment of methyl sulfone-induced contact inhibition under hypoxia implies that hypoxic cells also became cell cycle arrested, had inhibited DNA synthesis, became growth anchorage-dependent and had stopped migrating–all steps necessary for establishment of contact inhibition.

We define contact inhibition as well as the other functions listed above from our previous studies [3–5] as macrophenotypes [57] because establishment and maintenance requires coordinated regulation of several signaling pathways (e.g., HIPPO pathway, Wnt pathway, MAPK pathway) as well as regulation of specific proteins (e.g., E-cadherin, N-cadherin [4]). We show that methyl sulfone regulated expression of several metabolic proteins and miR-210 in the presence or absence of hypoxia in support of normal cell function. We define differential expression of metabolic proteins and miR’s as microphenotypes, and we show here that methyl sulfone induced expression of microphenotypes associated with four major functional activities in cells: aerobic glycolysis, angiogenesis, Fe-S metabolism and microtubule dynamics. Furthermore, several different enzymes, proteins and miR-210 involved with unique steps of aerobic glycolysis and Fe-S metabolism were examined showing that not just the first steps in these functional activities were altered by methyl sulfone; in other words, effects of methyl sulfone followed well-known cascades of these functional activities. The increase in ferroportin in methyl sulfone treated cells may provide an example of a cascading effect. Ferroportin is the only known efflux pump for iron recycling from cells. Dietary iron is absorbed, utilized, degraded and reutilized. When there is an excess of iron, ferroportin is degraded by the induction of the hormone, hepcidin, thereby slowing the recycling of systemic iron. If ferroportin remains low, cells increase iron, eventually causing iron overload. DNA synthesis is required for cell proliferation. Iron is required for ribonucleotide reductase, an enzyme that catalyzes DNA synthesis. It is known that cancer cells require higher than normal levels of iron and ribonucleotide reductase to support increasing cell proliferation [58]. One possible explanation for the presumed decease in cell proliferation when cancer cells are treated with methyl sulfone is the increase in ferroportin. The goal of treatment is to increase systemic iron and decrease cellular iron. When ferroportin is low the cell has access to increasing amounts of iron thereby supporting DNA synthesis.

Comparison of HIF-1α, PKM2 and ISCU1/2 protein expression in metastatic breast cancer cells and metastatic melanoma cells in the presence and absence of methyl sulfone and hypoxia reveals relatively similar patterns of expression. These data suggest that methyl sulfone is utilizing the same mechanisms in different types of metastatic tumors and under different microenvironments. Methyl sulfone also has similar dramatic effects on microtubules in these two types of metastatic cell lines in addition to T-cell leukemic lymphocytes.

### Multi-targeting by methyl sulfone versus single-targeting therapy

In metastatic breast cancer and melanoma cells, expression of four of the proteins affected by methyl sulfone (HIF-1α, PKM2, GLUT1 and VEGF) was hypoxia-dependent suggesting that at least initial regulation of these proteins by methyl sulfone occurred via HIF-1α. However, expression of four other proteins (LDHA, ISCU1/2, ferroportin and transferrin) and miR-210 were not hypoxia-dependent suggesting a non-HIF-1α mechanism of regulation by methyl sulfone. Methyl sulfone also had a major rapid impact on microtubules and tubulin, which may be independent of regulation of the eight proteins. In addition, literature search demonstrates that regulation of these eight proteins, whether initiated by HIF-1α or not, may involve at least a dozen different signaling pathways (Table 1).

**Table 1:** Comparison of methyl sulfone-affected proteins and signaling pathways that likely play a role in these proteins’ functions.

Methyl sulfone-affected proteins	References for methyl sulfone affected-proteins	Some signaling pathways that use proteins affected by methyl sulfone
HIF-1α	This study	● HIF
PKM2	This study	● Akt/β-catenin/Myc/Ras
GLUT1	This study	● Akt/mTOR/Myc/Ras
LDHA	This study	● Kruppel-like factor-4/PI3K/Myc/Ras
Transferrin	This study	● mTOR/MAPK/PI3K/Src
ISCU1/2	This study	● miR-210/p38/MAPK
Ferroportin	This study	● BMP-hepcidin/SMAD
VEGF	This study	● p38/Akt/MAPK/Fak-paxillin/PI3K/Ras
Tubulin/MTs	This study	● HIF/Stat3-stathmin/GSK-3β-Tau
E-cadherin	Caron et al., 2013a	● HIPPO/MAPK/Wnt
N-cadherin	Caron et al., 2013a	● Akt/β-catenin/Wnt
CD-44 (HCAM)	Caron et al., 2013b	● HIPPO/Jak-Stat/Wnt
OCT3/4	Caron et al., 2013b	● Akt/Jak-Stat/Hedgehog/MAPK/PI3K
SMA	Caron et al., 2013b	● Notch signaling

Proteins affected by methyl sulfone and their associated references are shown in column 1 and column 2 (SMA; myoepithelial smooth muscle actin). Signaling pathways that involve these proteins are shown in column 3. Pathways that have a commonality are separated by slashes. In some cases multiple distinct signaling pathways are involved with specific targets (e.g., VEGF, CD-44, OCT3/4); these pathways are separated by hyphens. This list of signaling pathways is not meant to be all-inclusive.

**Fig 6:**
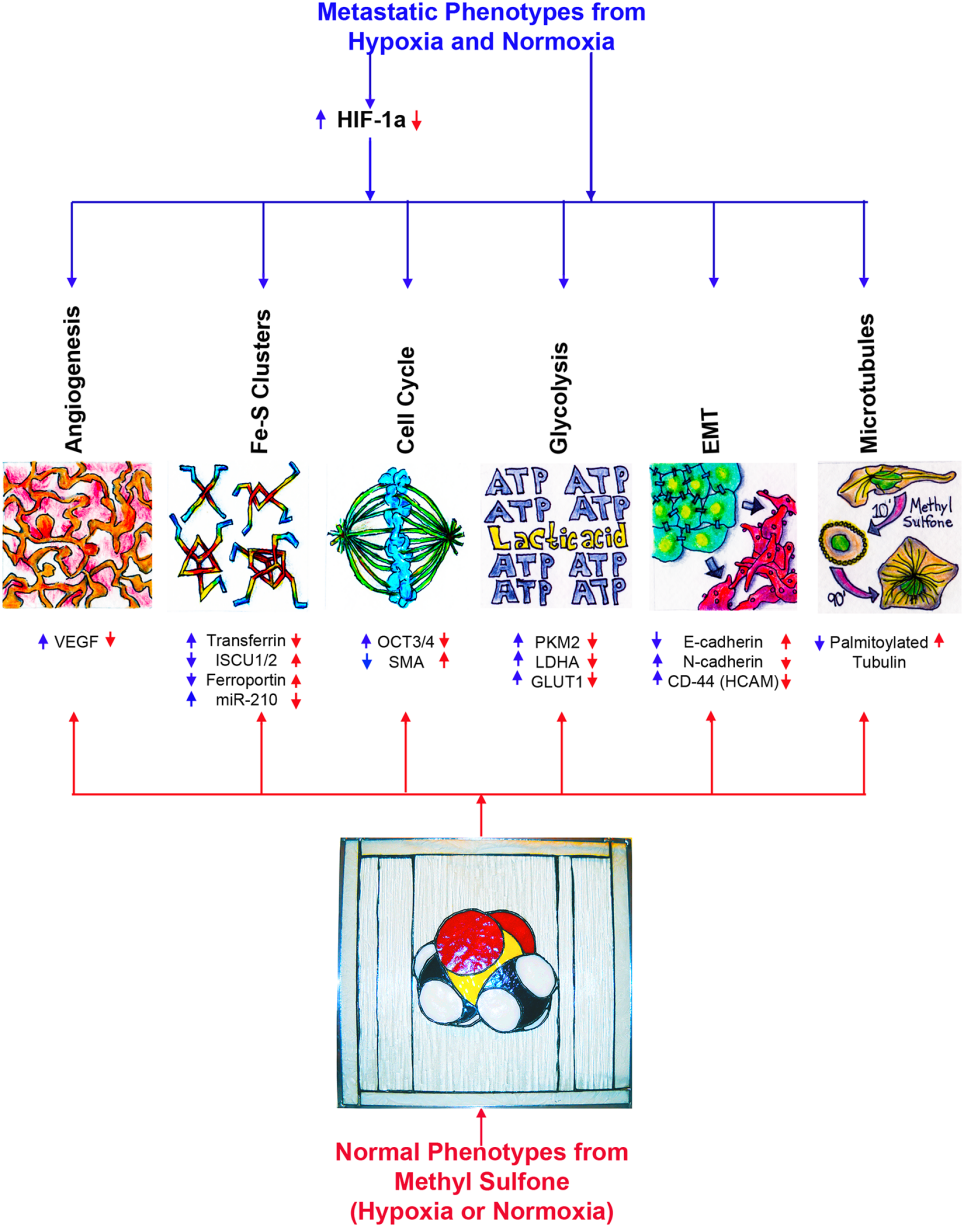
A model for cellular functions of metastatic cancer cells that are altered by methyl sulfone. Blue arrows represent the effect of metastasis on cellular functions found in metastatic cancer cells. Red arrows represent the effect of methyl sulfone on these functions in metastatic cancer cells. SMA (myoepithelial differentiation protein, Smooth Muscle Actin), E-cadherin and N-cadherin [4]; CD44 (HCAM) and OCT3/4 [5]; all remaining proteins and miR-210 are from this study. The methyl sulfone 3D chemical model is a stained glass piece created by JaMC.

Taken together, these data suggest that methyl sulfone has multiple intracellular targets to up-regulate and down-regulate expression and function of proteins, miR-210 and microtubules. Fig 6 is a model of macrophenotypes (angiogenesis, Fe-S cluster biogenesis, cell cycle, glycolysis, epithelial-mesenchymal transition (EMT), microtubule dynamics) and microphenotypes (VEGF, transferrin, ISCU1/2, ferroportin, miR-210, OCT3/4, SMA (myoepithelial differentiation protein, Smooth Muscle Actin) PKM2, LDHA, GLUT1, E-cadherin, N-cadherin, CD-44, palmitoylated tubulin) that are altered by methyl sulfone in the presence or absence of hypoxia. We are currently identifying specific signaling pathways that involve methyl sulfone.

Over time hypoxia induces resistance to the beneficial effects of many current chemotherapeutic drugs and radiation therapy [59,60]. On the other hand, it seems unlikely that metastatic cancer cells would become resistant to methyl sulfone. For example, we show here that a second round of hypoxia did not block formation of contact inhibition in metastatic cells. One possibility for lack of resistance in methyl sulfone-treated cells is that is that methyl sulfone appears to be multi-targeting. In single-targeting therapy, eventual resistance is a common phenomenon. Another possibility for lack of resistance with methyl sulfone is that changes associated with loss of metastatic phenotypes and re-emergence of normal phenotypes make resistance in “normal” cells a contradiction in terms.

All of the proteins and miR described in this study (HIF-1α, PKM2, LDHA, GLUT1, VEGF, transferrin, ISCU1/2, ferroportin, miR-210) are currently being investigated elsewhere for single-target chemotherapy with the goal of complete knockout of the specific proteins/miR. VEGF is an example of ongoing single-target research. The goal is to prevent vascularization of tumors by specifically blocking production of VEGF. However, recent studies have shown that complete knockout of VEGF leads to more aggressive tumors and a worse survival rate [61]. Additionally, single-target anti-HIF-1α therapeutics has demonstrated life-threatening problems with physiological responses to other diseases such as cardiac ischemia, renal disease and irritable bowel syndrome [62–64]. All of these single targets are normal proteins with bone fide functions that are required for daily living. Thus it is understandable that to completely block expression of one of these proteins would be detrimental to the individual. On the other hand, with the possible exception of PKM2 in M3 melanoma cells, methyl sulfone did not completely block expression of these proteins. It is possible that methyl sulfone fine-tunes expression of these proteins in response to normal cells needs.

### Effect of methyl sulfone on microtubules and tubulin

Methyl sulfone caused a rapid depolymerization of microtubules, palmitoylation of tubulin and association of tubulin with the plasma membrane. No known microtubule drug (e.g., Taxol, vinblastine, nocodazole, colchicine) has this effect. Microtubules may be completely depolymerized by known microtubule drugs, but the tubulin does not adhere to the inner surface of the plasma membrane. At high concentrations, depolymerized cytosolic tubulin is lethal to cells, and will send cells into apoptosis. This lethality is seen in both normal and malignant cells. Cells do autoregulate cytosolic tubulin levels by degrading excess tubulin [65], but in the presence of known microtubule drugs, complete depolymerization of microtubules overwhelms the autoregulation system, and the cells die.

One possibility to explain why cells do not die in the presence of methyl sulfone is that palmitoylated tubulin on the plasma membrane prevents the cytotoxic effects of cytosolic tubulin. The plasma membrane may be analogous to a storage unit for depolymerized tubulin. In addition, palmitoylation is a reversible post-translational modification. After a couple of hours in methyl sulfone, microtubules in cells begin to reform. We propose that tubulin is likely de-palmitoylated, released from the plasma membrane and once again becomes available for microtubule polymerization in a pattern specified by normalizing parameters of the methyl sulfone-treated cell.

Finally, microtubule assembly is exquisitely sensitive to even slight changes in the tertiary structure of tubulin [66]. However, a 50-fold molar excess of methyl sulfone (2 mM) to microtubule protein (40 μM) had no effect on microtubule assembly. This suggests that methyl sulfone does not alter the tertiary structure of tubulin.

### Lack of toxicity of methyl sulfone and possible use in clinical trials

Toxicity (LD50) in mammals is defined as the concentration of a substance that causes the death of 50% of the animals, usually within 14 days. In 1984, Gosselin [67] established a table to display relative levels of LD50 between compounds. See Gosselin’s Table in S2 Table: LD50 toxicity rating for humans (70 KG body weight). In Gosselin’s Table the most toxic molecule (<5 mg/kg) is termed “super toxic”; the least toxic (>15 g/kg) is termed “practically nontoxic”. For methyl sulfone, an accurate LD50 has not yet been determined because studies have used concentrations as high as 20 g/kg and no animals died, nor did any of the animals have adverse events [68]. In addition, studies with rats showed that methyl sulfone is not a teratogen [69]. Therefore, we can assume that the LD50 for methyl sulfone is >20 g/kg. To put this number in perspective, the LD50 for water, which is considered the least toxic substance on earth, is approximately 90 g/kg. Conversely, the “super toxic” non-water soluble chemotherapeutic drug Taxol has an LD50 of 1.5 mg/kg. The LD50 for the common drug, aspirin, is 1.7 g/ml and is therefore classified by the Gosselin scale as “moderately toxic”.

Methyl sulfone’s high, “practically nontoxic” LD50, and methyl sulfone’s granted GRAS status from the FDA on July 11, 2007, makes this molecule particularly well suited for safe treatment of cancer patients–except for one remaining issue. In our studies we examined different metastatic cell lines and human normal and cancerous breast tissues. We consistently find that 200 mM methyl sulfone is the optimal concentration to cause metastatic cells to revert to normal cells. The question is, “Is it possible to treat cancer patients with a concentration of 200 mM methyl sulfone?” This concentration, 200 mM, seems high, when compared to standard of care treatment compounds, such as Taxol above.

The answer lies in the fact that methyl sulfone is highly water-soluble (>150 mg/ml). A one-liter 5X methyl sulfone solution (1M) can easily be made in any water-based solvent used in infusions. Assuming an adult male contains 5 liters of blood, an infusion of 1 liter of 1M methyl sulfone will lead to 200 mM methyl sulfone in the patient’s blood stream.

In conclusion, our data demonstrate that methyl sulfone normalizes hypoxic and non-hypoxic expression of metabolic enzymes, proteins and miR-210 that are associated with the metastatic phenotype. These changes involve perhaps as many as a dozen different signaling pathways, including ones that involve microtubules, suggesting that methyl sulfone is a multi-targeting molecule. Methyl sulfone is also one of the least toxic molecules known to man, and is highly soluble in water making this molecule ideal for clinical trials.

## Supporting Information

S1 Table In vitro microtubule assembly in the presence and absence of methyl sulfone. (DOCX)

S2 Table LD50 toxicity rating for humans (70 KG body weight). (DOCX)
