# Supplementary material for: Methyl Sulfone Blocked Multiple Hypoxia- and Non-Hypoxia-Induced Metastatic Targets in Breast Cancer Cells and Melanoma Cells
Source: PLoS One. 2015 Nov 4;10(11):e0141565. doi: 10.1371/journal.pone.0141565 (PMC4633041; doi:10.1371/journal.pone.0141565)
Supplement: S1 Table — (DOCX) [file pone.0141565.s001.docx]

**Supporting Material: S1 Table**

**S1 Table: In vitro microtubule assembly in the presence and absence of methyl sulfone**

| **Methyl Sulfone** |  | **0** | **0.8μM** | **4μM** | **40μM** | **400μM** | **2mM** | **_** | **4μM** |
| --- | --- | --- | --- | --- | --- | --- | --- | --- | --- |
| **Taxol** |  | **-** | **-** | **-** | **-** | **-** | **-** | **4μM** | **4μM** |
| **MTP** |  | **40μM** | **40μM** | **40μM** | **40μM** | **40μM** | **40μM** | **40μM** | **40μM** |
|  |  |  |  |  |  |  |  |  |  |
| **Vmax** | **A** | 5.0 | 7.6 | 5.9 | 5.7 | 4.8 | 4.9 | 12.3 | 17.8 |
|  | **B** | 5.3 | 5.8 | 4.9 | 6.2 | 5.1 | 6.0 | - | - |
|  |  |  |  |  |  |  |  |  |  |
| **AUC** | **A** | 1572 | 1705 | 1560 | 1599 | 1565 | 1632 | 2076 | 2035 |
|  | **B** | 1565 | 1573 | 1565 | 1654 | 1554 | 1593 | - | - |
|  |  |  |  |  |  |  |  |  |  |
| **Δ-OD_340_** | **A** | 0.194 | 0.226 | 0.204 | 0.199 | 0.197 | 0.209 | 0.253 | 0.246 |
|  | **B** | 0.204 | 0.196 | 0.202 | 0.230 | 0.203 | 0.203 | - | - |
|  |  |  |  |  |  |  |  |  |  |
| **Lag Time**  (min) | **A** | 4.0 | 4.0 | 4.0 | 3.3 | 3.3 | 3.3 | 0 | 0 |
|  | **B** | 3.3 | 3.3 | 3.3 | 3.3 | 4 | 4 | - | - |

**MTP:** microtubule protein; **Vmax:** maximum rate of the microtubule growth phase; **AUC:** area under the curve; **Δ-OD:** change in OD_340_ over 90 min; **A and B:** duplicates in 96-well plate.
