# Supplementary material for: Methyl Sulfone Blocked Multiple Hypoxia- and Non-Hypoxia-Induced Metastatic Targets in Breast Cancer Cells and Melanoma Cells
Source: PLoS One. 2015 Nov 4;10(11):e0141565. doi: 10.1371/journal.pone.0141565 (PMC4633041; doi:10.1371/journal.pone.0141565)
Supplement: S2 Table — (DOCX) [file pone.0141565.s002.docx]

**Supporting Material: S2 Table**

| **S2 Table: LD50 toxicity rating for humans (70 KG body weight)** | |
| --- | --- |
| Rating/Class | Dose |
|  |  |
| 6. Super toxic | <5 mg/kg |
| 5. Extremely toxic | 5–50 mg/kg |
| 4. Very toxic | 50–500 mg/kg |
| 3. Moderately toxic | 0.5–5 g/kg |
| 2. Slightly toxic | 5–15 g/kg |
| 1. Practically nontoxic* | >15 g/kg* |
|  |  |
| **Reference:** Gosselin, et al. (1984). *Clinical Toxicology of Commercial Products.*  Baltimore: Williams & Wilkens (Reference 67). | |

*1. “Practically nontoxic” includes methyl sulfone and water.
